# Extracompartmental Two-Injection Technique for Treating De Quervain Tenosynovitis

**DOI:** 10.7759/cureus.102956

**Published:** 2026-02-04

**Authors:** Tyler S Iodence, William F McCormick, Jeffrey A Marchessault

**Affiliations:** 1 Orthopedic Surgery, Watauga Orthopaedics, Bristol, USA; 2 Orthopedic Surgery, East Tennessee State University, Johnson City, USA

**Keywords:** corticosteroid injection, de quervain, hand, injection, tenosynovitis

## Abstract

In this study, we prospectively analyzed patients diagnosed with and treated for De Quervain tenosynovitis (DQT) to evaluate the efficacy of a novel extracompartmental two-injection technique. Fifteen patients completed a Visual Analog Scale for pain, grip strength testing, and physical examination before receiving injections. After four weeks, these same measurements were repeated and compared to baseline. Statistical analysis was conducted using two-tailed t-tests to compare symptoms before and four weeks after treatment, pain associated with each injection, and grip strength before and four weeks after treatment. Results suggest that the extracompartmental two-injection technique led to significant symptom improvement and absent physical exam findings in 100% of patients, complete symptom resolution in 73% of patients, and improved grip strength in 93% of patients with DQT. We describe this reproducible technique to help providers and patients avoid the pain and risks associated with intracompartmental steroid injection while still achieving short-term outcomes comparable to those reported with other injection techniques.

## Introduction

De Quervain tenosynovitis (DQT) is a painful condition caused by inflammation and thickening of the tendon sheath and overlying retinaculum of the first dorsal compartment tendons of the wrist [1]. The prevalence is estimated at 1.3% in women and 0.5% in men, typically presenting in the fourth and fifth decades of life [2-4]. Originally thought to result solely from inflammation, histological studies have demonstrated intrinsic degeneration and thickening of the tendon sheath secondary to the accumulation of mucopolysaccharides within the tissue [3,5]. This subsequent narrowing of the canal creates a restrictive environment for the abductor pollicis longus (APL) and extensor pollicis brevis (EPB) tendons to glide through the sheath [6,7].

Diagnosis of DQT can be made clinically using maneuvers such as the Finkelstein test and the Eichhoff test, which tension the first compartment tendons and have sensitivities of 100% and 89%, respectively [8]. Additional imaging, such as ultrasonography demonstrating thickening of the tendon sheath, can help distinguish DQT from other wrist pathologies, including intersection syndrome or carpometacarpal arthritis [9].

Treatment begins conservatively with activity modification and corticosteroid injections [10], which have been shown to effectively treat the disease in up to 90% of cases [11]. For refractory cases, surgical release of the first dorsal compartment remains an option [12].

Failure of conservative management is thought to result from anatomic variants of the first dorsal compartment, including entirely separate sheaths or a septum dividing the sheath into two distinct subcompartments [13,14]. Found in 40-60% of patients, this division can sequester the injected corticosteroid in a single subcompartment [13], preventing adequate medication delivery to both diseased tendons [13,14].

Corticosteroid injections can be acutely painful for many patients due to factors such as anatomical variation, degree of sheath constriction, extent of inflammation, needle size, injection speed and volume, and patient sensitivity to pain [15]. We hypothesize that a significant portion of injection-related pain results from injecting directly into the compartment, thereby increasing compartment pressure and exacerbating the underlying source of pain associated with DQT.

Injection techniques can address many of these factors through needle selection, injection speed, and the use of ethyl chloride spray; however, techniques have historically relied on single, intracompartmental injections [15]. Danda et al. describe a two-injection technique that successfully treated patients with variant, divided compartments, although this approach required ultrasound guidance and intracompartmental injection [16]. To reduce the need for ultrasound and minimize pain associated with intracompartmental injection, the senior author (JAM) developed an extracompartmental injection technique for the first dorsal compartment tendons. We empirically studied this technique to document its effectiveness in providing short-term pain relief.

This technique first employs a small, 27-gauge needle for injection into the subfascial space proximal to the retinaculum. A second, smaller dose of steroid is then administered distal to the retinaculum to deliver medication to the sometimes-isolated EPB tendon. By injecting both proximal and distal to the retinaculum, the steroid can passively spread along the tendon and sheath without increasing intracompartmental pressure, ensuring adequate coverage of the diseased area regardless of subcompartments or the presence of a septum. Local anesthetic administered with the first injection improves steroid diffusion [17] and is expected to reduce pain associated with the second injection.

Although steroid injections have been shown to produce favorable outcomes [11], limited evidence exists regarding optimization of injection techniques. We examined patients undergoing corticosteroid injection for DQT before and after the novel two-injection technique to determine its efficacy and to assess pain associated with the procedure itself. The aim of this study is to present our technique, evaluate its effectiveness in treating DQT, and report four-week outcomes in hopes of improving the patient experience during injections. We hypothesize that the newly described technique will provide symptom relief at rates equivalent to or greater than those reported in the literature (up to 90%) [11]. We further hypothesize that a local anesthetic administered with the first injection will decrease the pain associated with the second injection.

## Materials and methods

From 2020 to 2023, a single provider evaluated patients with radial-sided wrist pain in an orthopedic hand surgery clinic. After a thorough history and physical examination, patients diagnosed with DQT were offered conservative management, including the option of a corticosteroid injection.

Exclusion criteria included a history of prior injections, trauma, or surgery to the hand or wrist; CMC joint tenderness; pregnancy; diabetes; glaucoma; and a BMI greater than 35. Informed consent was obtained, explaining the study and procedure in detail, and participants were given the opportunity to ask questions.

Physical examination

Prior to treatment, patients completed a Visual Analog Scale (VAS), rating their pain with gentle passive flexion of the thumb with ulnar deviation (Finkelstein test) and active ulnar deviation of the wrist while gripping the thumb in the palm (Eichhoff test). Grip strength was measured using a dynamometer, with the average of three maximal efforts recorded for each patient.

Treatment

Injection sites were first cleaned with alcohol. The first syringe, labeled “proximal,” was filled with 10 mg of Kenalog and 1 cc of 0.25% Marcaine without epinephrine. The second syringe, labeled “distal,” was filled with 5 mg of Kenalog and 0.5 cc of 0.25% Marcaine without epinephrine. Twenty-seven-gauge needles were used for both injections.

**Figure 1:**
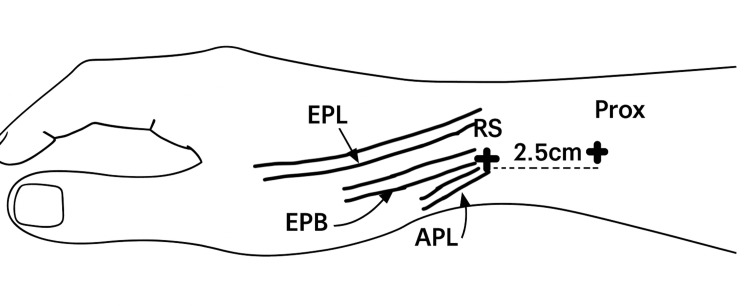
Depiction of injection sites and relevant tendon anatomy on the right hand Notable features include the RS, Prox, EPL, EPB, and APL. APL, abductor pollicis longus; EPB, extensor pollicis brevis; EPL, extensor pollicis longus; Prox, proximal injection site; RS, radial styloid

A single provider injected all patients using the same novel two-injection technique described here. The provider palpated the distal aspect of the radial styloid and measured 2.5 cm proximal to the styloid to indicate the proximal end of the retinaculum (Figure 1). With the wrist in a neutral resting position, the tendons of the first dorsal compartment were palpated, identifying the radial and ulnar borders of the tendons proximal to the retinaculum. The needle was then inserted between the two tendons until bone was felt and then slightly retracted prior to injection. The provider ensured there was no resistance during injection, as this could indicate intratendinous placement. The provider then waited five minutes before administering the second injection.

For the second injection, the provider palpated the tip of the radial styloid, indicating the distal extent of the retinaculum, and marked the injection site just distal to the styloid (Figure 1). The superficial branch of the radial nerve was gently displaced from the site using radial traction on the skin while palpating the extensor tendons. The injection was administered deep to the subcutaneous layer, again confirming no resistance to ensure the injection was extratendinous. Finally, patients completed a VAS documenting pain associated with each injection. No activity restrictions were given, and patients were instructed to follow up after four weeks.

At the four-week follow-up appointment, patients were reexamined using the same measures and instruments as those used prior to the procedure. This included completing a VAS for pain with the Finkelstein and Eichhoff tests, as well as grip strength testing. All findings and measurements were recorded in a Microsoft Excel spreadsheet.

Pre- and post-procedure measurements were compared using two-tailed t-tests. Similarly, VAS scores for pain associated with the first and second injections were compared using t-tests.

## Results

**Figure 2:**
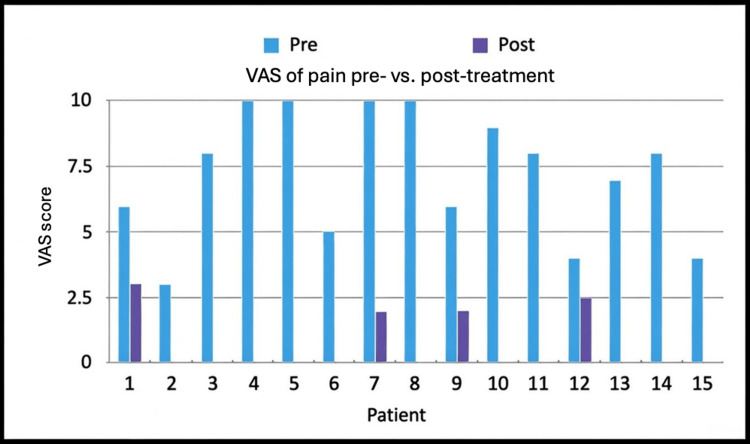
Patient-reported pain prior to (pre) and following (post) treatment Pain was assessed using a VAS ranging from 0 to 10. A two-tailed t-test comparing the averages of the two groups yielded a p-value of 0.0000004. VAS, Visual Analog Scale

From 2020 to 2023, 15 patients were treated with injections and met the criteria for review. At the four-week follow-up, there was a statistically significant decrease in reported pain. All patients (100%) experienced a reduction in pain, and 11 patients (73%) had complete resolution of their pain. As shown in Figure 2, the average VAS score prior to injection was 7.2/10, compared to 0.63/10 at the four-week follow-up. This represents a statistically significant decrease in pain (p = 0.0000004).

**Figure 3:**
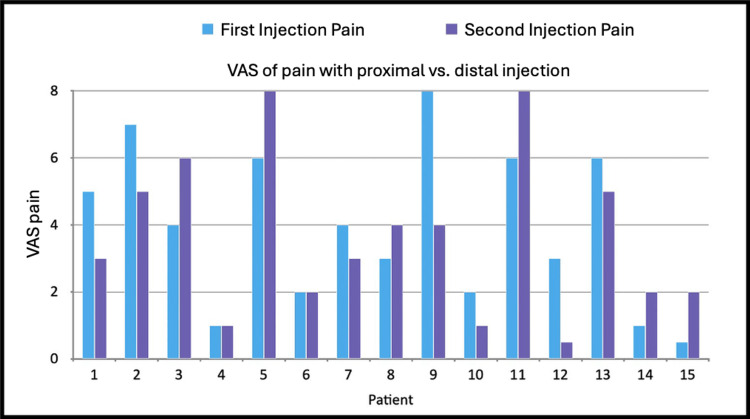
Patient-reported pain for the first (proximal) and second (distal) injections Pain was assessed using a VAS ranging from 0 to 10. A two-tailed t-test comparing the averages of the two groups yielded a p-value of 0.58. VAS, Visual Analog Scale

The average reported pain associated with the first (proximal) injection was 3.9/10, while the second (distal) injection averaged 3.6/10. Seven patients reported less pain with the second injection compared to the first, six reported greater pain with the second injection, and two reported the same pain for both injections (Figure 3). There was no statistically significant difference in pain between the two injections (p = 0.58).

**Figure 4:**
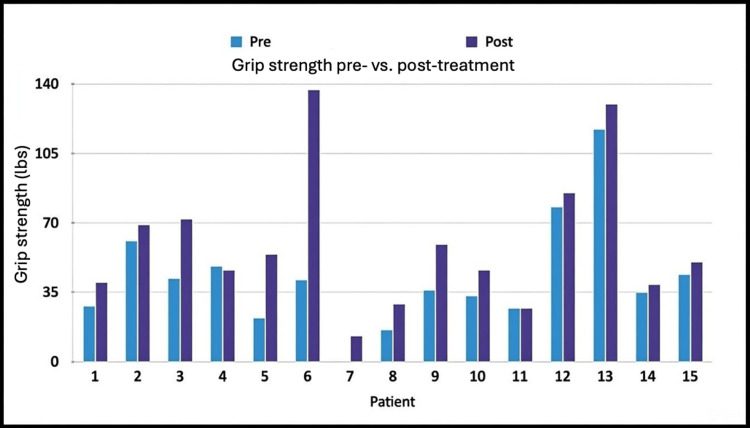
Grip strength in pounds (lbs) as measured by dynamometer prior to treatment (pre) and four weeks following treatment (post) A two-tailed t-test comparing the averages of the two groups yielded a p-value of 0.011.

Fourteen of 15 patients (93%) demonstrated increased grip strength at the four-week follow-up. The average grip strength prior to treatment was 41.8 lbs, which increased to 60.2 lbs at follow-up (Figure 4). A two-tailed t-test comparing grip strength before treatment and at four weeks yielded a p-value of 0.011, indicating a statistically significant improvement.

Finally, prior to treatment, all 15 patients (100%) demonstrated a positive Finkelstein test. At four-week follow-up, all patients demonstrated a negative Finkelstein test, indicating clinical improvement.

## Discussion

Our study demonstrates that an extracompartmental two-injection technique effectively reduces or resolves DQT symptoms, including radial-sided wrist pain, at four-week follow-up. The observed success rates are consistent with those reported in the literature [5,10,11,13,18-20]. This technique led to symptom reduction in 100% of patients and complete symptom resolution in 73% of patients at four weeks. Over the same period, 93% of patients demonstrated increased grip strength. While the provider noted improved patient comfort with this technique, we did not observe that the first injection reduced pain associated with the second injection, likely because the local anesthetic did not reach the superficial nerves distal to the retinaculum.

Conservative management of DQT with corticosteroid injections has a well-documented record of successfully treating radial-sided wrist pain [11]. Many previously described techniques recommend injection directly into the tendon sheath [2,13,16,18,21,22]; however, we believe this can cause significant discomfort by increasing intracompartmental pressure around the diseased tendon and sheath. Additionally, current literature suggests that poor response to intracompartmental steroid injections is often due to anatomic variants, such as separate sheaths or a septum, preventing adequate medication spread to both tendons [2,13,14,21].

In a prospective double-blind study, Zingas et al. injected dye along with a steroid into the first dorsal compartment and radiographed patients’ wrists to evaluate dye spread [21]. They found that most patients with suboptimal treatment responses had no dye observed in the EPB compartment. They also noted cases in which dye failed to reach both the APL and EPB in patients with normal anatomy (both tendons in the same compartment), which they attributed to poor injection technique.

Mirzanli et al. examined the accuracy of intracompartmental injections in cadavers by injecting dye followed by dissection of the dorsal wrist [2]. They found that 28% of injections demonstrated inadequate spread of the medication due to the presence of a septum. They concluded that injections should be performed separately over the two tendons to account for the possible presence of a septum within the compartment.

Many techniques have been introduced in response to these findings to improve outcomes. Two studies demonstrated favorable results using ultrasound guidance. Bing et al. implemented an ultrasound-guided injection strategy in patients with known anatomic variations and observed successful pain reduction in 22 of 23 patients [22]. Kamel et al. also used ultrasound to target the EPB in patients with septations and reported a significant decrease in injection-associated pain compared to manual injections [9].

Our technique addresses the shortcomings and the need for ultrasound identified by these studies. By directing injections both proximal and distal to the retinaculum and any possible septation, significant increases in compartmental pressure are avoided, and the medication can adequately spread to the diseased area even in the presence of subcompartments.

This study has several limitations, including a small sample size of 15 patients, a lack of a control group, and only four weeks of follow-up. Although our findings support the efficacy of this technique for managing DQT, further research is needed to determine whether increasing the interval between injections could reduce pain with the second injection. Additionally, future studies should compare acute pain during extracompartmental injections to intracompartmental injections in a larger control group to optimize patient comfort during these procedures.

## Conclusions

Based on these results, this extracompartmental two-injection technique effectively delivers steroids to the diseased area without requiring direct injection into the tendon sheath, which we hypothesize is a major contributor to injection-associated pain. At follow-up, patients reported improvement or complete resolution of pain, demonstrated negative physical exam findings, and showed increased grip strength. There was no significant difference in injection-associated pain between the two injections. We believe this technique is easily reproducible and allows providers to effectively treat DQT with corticosteroids while enhancing patient comfort and avoiding the additional cost or time associated with ultrasound guidance.
